# Gender differences in school achievement: The role of self-regulation

**DOI:** 10.3389/fpsyg.2013.00442

**Published:** 2013-07-17

**Authors:** Mirjam Weis, Tobias Heikamp, Gisela Trommsdorff

**Affiliations:** Department of Psychology, Developmental and Cross-Cultural Psychology, University of KonstanzKonstanz, Germany

**Keywords:** self-regulation, gender differences, school achievement, behavior regulation, emotion regulation

## Abstract

This study examined whether different aspects of self-regulation (i.e., emotion and behavior regulation) account for gender differences in German and mathematics achievement. Specifically, we investigated whether higher school achievement by girls in comparison to boys can be explained by self-regulation. German and mathematics achievement were assessed in a sample of 53 German fifth graders (19 boys, 34 girls) using formal academic performance tests (i.e., reading, writing, mathematics) and teachers' ratings (i.e., grades in German and mathematics). Moreover, teachers rated children's behavior regulation using the Self-Control Scale (SCS-K-D). Children's self-reported strategies of emotion regulation were assessed with the Questionnaire for the Measurement of Stress and Coping in Children and Adolescents (SSKJ 3-8). Age and intelligence (CFT 20-R) were included as control variables. Analyses of mean differences showed that girls outperformed boys in German achievement and behavior regulation. Regression analyses, using a bootstrapping method, revealed that relations between gender and German achievement were mediated by behavior regulation. Furthermore, we found a suppression effect of behavior regulation on the relation between gender and mathematics achievement: boys' mathematics achievement was underestimated when the analyses did not control for behavior regulation. We discuss these results from a developmental perspective and within the theoretical framework of self-regulation and achievement.

Currently, both scientific literature and German mass media are discussing the discrepancy in school achievement between boys and girls, going so far as to call boys the new losers of the educational system (Spiewak, 2010, August 5). Several studies have found significant gender differences in school achievement favoring girls over boys (Cole, 1997; Duckworth and Seligman, 2006). According to the German census, there are more girls than boys in higher secondary schools, whereas more boys than girls attend lower secondary schools. As a consequence, more girls achieve the general qualification for university entrance, whereas more boys complete the certificate of lower secondary school (Statistisches Bundesamt, 2011).

The reasons for these gender differences in school achievement have not been clarified yet. Past research has shown that besides cognitive abilities (e.g., intelligence; Deary et al., 2007; Spinath et al., 2010) the motivation and ability to self-regulate is positively associated with school achievement (Duckworth and Seligman, 2005; Suchodoletz et al., 2009). In line with these findings, previous studies have indicated that specific components of self-regulation—behavioral regulation or self-regulated learning—could contribute to gender differences in school achievement (Duckworth and Seligman, 2006; Kuhl and Hannover, 2012). However, by only investigating behavior regulation, these previous studies neglected the wider conceptualization of self-regulation. The concept of self-regulation includes both behavior regulation and emotion regulation, and both aspects of self-regulation may be related to children's school achievement (Blair, 2002; Calkins, 2007; McClelland et al., 2007). Therefore, it is important to understand the contribution of behavior and emotion regulation to gender differences in school achievement.

In the present study, we investigated in a sample of German fifth graders who had just transitioned from primary school to secondary school whether self-regulation mediates effects of gender on school achievement. In particular, we studied the relations between different aspects of self-regulation (i.e., behavior regulation, emotion regulation) and school achievement in different domains (i.e., German and mathematics achievement).

## Gender differences in school achievement

Past research suggested that girls are in general more successful in school than boys. Hartley and Sutton (2013) have recently reported that especially boys develop gender stereotypes according to which girls are perceived as academically superior with regard to motivation, ability, performance, and self-regulation. However, previous studies revealed rather inconsistent results concerning gender differences in different domains of school achievement. In the present study, we focused on achievement in German and mathematics because performance in these subjects is seen as an important aspect of school achievement (Schrader and Helmke, 2008). Previous large-scale studies revealed higher German achievement by girls in comparison to boys (Stanat and Kunter, 2003; Stanat et al., 2012). However, the picture of gender differences in mathematics achievement is less clear (Hannover and Kessels, 2011; Stanat et al., 2012). While in some studies boys exceeded girls in mathematics achievement, in other studies no gender differences in mathematics achievement were found (Hannover and Kessels, 2011). For instance, Machin and Pekkarinen (2008) argued that mixed evidence for gender differences in school achievement could be explained in part by a higher variance of boys' in comparison to girls' school achievement.

As Hyde (1990) pointed out, meta-analyses have consistently shown that there are no significant gender differences in general cognitive abilities. Thus, although cognitive abilities are significantly and positively related to school achievement, they cannot explain gender differences in school achievement (Spinath et al., 2010). Therefore, further “non-cognitive” variables have been examined in an attempt to explain gender differences in school achievement. For instance, Spinath et al. (2010) highlighted the importance of personality and motivation for gender differences in school achievement. They found that a higher level of extraversion was associated with higher grades for girls but lower grades for boys. Pomerantz et al. (2002) noted that girls want to please adults to a higher degree than do boys, which leads to girls' higher school grades. Furthermore, stereotypes are an important influence on school achievement in that negative stereotypes disrupt girls' mathematics performance (e.g., Keller and Dauenheimer, 2003). However, a rarely considered explanation for gender differences in school achievement from a developmental point of view is *self-regulation* (Duckworth and Seligman, 2006).

## Self-regulation and school achievement

Various terms and definitions have been used to conceptualize self-regulation and its components (McClelland et al., 2010). Here, self-regulation is understood as the motivation and ability to maintain goal-directed actions over time and across several situational contexts in order to achieve desired goals (Karoly, 1993). Although relatively stable differences exist between individuals with regard to the motivation and ability to self-regulate (Raffaelli et al., 2005), there is situation specific variance in self-regulation within individuals depending on domain-specific temptation (Tsukayama et al., 2012). Self-regulation is conceived as a broad construct which includes the more specific components behavior regulation and emotion regulation. Behavior regulation includes the motivation and ability to pay attention, to follow rules, to resist temptation, and to inhibit inappropriate actions (e.g., McClelland et al., 2007; Heikamp et al., 2013). In contrast, emotion regulation is a process that serves to initiate, to inhibit, to maintain, or to modulate the experiences of emotions in order to achieve social adaptation or individual goals (Eisenberg and Spinrad, 2004). In the present study, we focused on strategies of emotion regulation that aim to change the experience of negative emotions (Cole et al., 2004). According to the transactional model of stress and coping, problem-oriented and emotion-oriented strategies can be distinguished (Lazarus and Folkman, 1984). Problem-oriented strategies are directed to the context and aim to change a situation that elicited negative emotions. In contrast, emotion-oriented strategies aim to regulate emotional experiences by changing the appraisal of a situation. Whereas problem-oriented strategies include instrumental actions that aim to change the cause of the negative emotional experience, emotion-oriented strategies involve the behavioral and cognitive avoidance of the problem (Lohaus et al., 2006; Skinner and Zimmer-Gembeck, 2007). Behavior regulation and emotion regulation can be seen as two distinct components of self-regulation. Even though behavior and emotion regulation are distinguishable concepts, they are interrelated during the course of development (Raffaelli et al., 2005). Considering the broad conceptualization of self-regulation and taking into account that self-regulation is a multidimensional construct (e.g., Duckworth and Kern, 2011), it is important to take a more nuanced perspective on self-regulation by viewing behavior regulation and emotion regulation as interrelated but separate aspects of self-regulation.

The transition from elementary to secondary school is associated with increasing demands such as self-organization, homework, and exam preparation in various subjects. Hence, children need to adopt self-regulated learning strategies (through goal-setting, strategy use, and self-monitoring) to be successful in school (Blair, 2002). Students have to develop self-regulation strategies, which include goal oriented processes that aim to regulate emotions and behavior in order to adapt successfully to school (Zimmerman, 1990; Schunk and Zimmerman, 1997; Suchodoletz et al., 2009). Self-regulation, with its components behavior regulation and emotion regulation, is positively associated with school achievement (Calkins, 2007; McClelland et al., 2007). According to Zimmerman and Schunk (2011) self-regulated students are effective in school because they set learning goals, apply effective learning strategies, monitor their own goal progress, establish a productive learning environment, and develop self-efficacy beliefs for learning.

Behavior regulation enables one to remember and follow instructions and to concentrate on tasks without getting distracted. Therefore, behavior regulation is positively related to the development of positive classroom behavior and academic achievement (McClelland et al., 2007). Most notably, behavior regulation accounts for additional variance in school achievement above and beyond the variance that is explained by intelligence (e.g., Duckworth and Seligman, 2005; Suchodoletz et al., 2009).

Blair (2002) argued that adequate emotion regulation in the classroom facilitates cognitive processes (e.g., memory, attention, planning, problem solving), which are necessary for scholastic learning. In the school context, emotions have to be regulated to allow for the child's appropriate achievement behavior (Trommsdorff, in press). In general, both problem-oriented and emotion-oriented strategies can be adaptive strategies to regulate emotions. It depends on the situation which strategy brings higher benefits (Lohaus et al., 2006). Adaptive emotion regulation means to adopt strategies flexible depending on the situation (Lohaus et al., 2006; Skinner and Zimmer-Gembeck, 2007). Regarding strategies which are used to regulate negative emotions in the school context, studies have shown that problem-oriented strategies have positive effects whereas emotion-oriented strategies (e.g., avoidance, distraction) have negative effects on school achievement (e.g., Brdar et al., 2006; Cohen et al., 2008). This effect can be seen in individual differences in preparing for examinations and in different relations with achievement in the school context. For instance, students who are more likely to use problem-oriented strategies prepare for examinations and plan their work, whereas students who use emotion-oriented strategies do not actively cope with the future examination and thus do not take enough time to study (Zeidner, 1995). Whereas problem-oriented strategies might be more effective for school achievement, emotion-oriented strategies might be adaptive in order to regulate emotions in the short term (e.g., to feel good) but may have negative consequences regarding school achievement in the long run.

## Gender, self-regulation, and school achievement

Bjorklund and Kipp (1996) argue that a greater evolutionary necessity of women to control their emotional and behavioral reactions in social situations has led to women's higher self-regulation abilities. Davis (1995) suggested that girls are more expected than boys to act according to social rules, which induces girls having more practice and therefore a better ability to regulate their behaviors and emotions. In line with this view, meta-analytic studies have shown that girls have a higher motivation and ability to engage in behavior regulation than boys (e.g., Silverman, 2003; Else-Quest et al., 2006; Cross et al., 2011). Gender differences have also been reported with regard to the habitual use of emotion regulation strategies. For instance, girls tend to use strategies that aim to solve a problem in order to feel better (i.e., problem-oriented strategies) more often than do boys. In contrast, boys tend to emotionally disengage from stressful situations (i.e., emotion-oriented strategies) more often than do girls (Eschenbeck et al., 2007).

Because (a) there is evidence for greater school achievement and self-regulation by girls and (b) self-regulation is positively related to school achievement, one may ask whether self-regulation accounts for gender differences in school achievement. In a sample of US-American eighth graders, Duckworth and Seligman (2006) found that girls' higher school achievement can be explained in part by behavior regulation. Kuhl and Hannover (2012) showed that in a sample of German fourth graders, teachers' ratings of children's self-regulated learning could partly explain gender differences in school achievement. Here, we examined both behavior regulation and emotion regulation as aspects of self-regulation. We investigated whether the relation between gender and school achievement (German and mathematics) is mediated by self-regulation (behavior regulation and emotion regulation). Further, we extended the mediation models by controlling for age and intelligence.

## Study aims

The present research aimed to test if gender differences in school achievement can be explained by gender differences in self-regulation. Therefore, two mediation models were tested to investigate whether behavior regulation and emotion regulation mediate the association between gender and school achievement in German and mathematics. In line with previous findings (e.g., Cole, 1997; Duckworth and Seligman, 2006), we hypothesized that girls have greater school achievement than do boys. Building on past research on gender-differences in behavior regulation (e.g., Silverman, 2003; Else-Quest et al., 2006; Cross et al., 2011), we expected that girls show a higher motivation and ability for behavior regulation than boys. Regarding gender differences in emotion regulation, we hypothesized that girls show problem-oriented strategies more often than boys, whereas boys show emotion-oriented strategies more often than girls (Eschenbeck et al., 2007). In order to extend the scope of previous studies, we examined whether different aspects of self-regulation (i.e., emotion and behavior regulation) account for gender differences in school achievement. Based on past findings, we expected that the relations between gender and school achievement are mediated by behavior regulation (Duckworth and Seligman, 2006; Kuhl and Hannover, 2012). In extension of past research, we investigated whether there is an indirect effect of gender on school achievement mediated by children's use of emotion regulation strategies (i.e., problem-oriented strategies, emotion-oriented strategies).

## Materials and methods

### Participants

Fifty-seven children participated in the study in summer 2010. The children attended 22 different fifth grade classes in seven different schools in a town in Southern Germany. The class teachers of the 22 fifth grade classes were asked to complete questionnaires about those children of their class who took part in the study. Number of students for whom each class teacher provided reports of grades and behavior regulation ranged from 1 to 5. Four children were excluded from data analysis because of incomplete data sets. Hence, the sample consisted of 53 fifth graders (34 girls) and their class teachers. Children's mean age was 11.23 years (*SD* = .54). Twenty-two (100%) class teachers (16 female, 6 male) completed questionnaires about the school achievement (i.e., grades) and behavior regulation of those students who attended their class. Thirty-nine (74%) mothers completed questionnaires on their highest school graduation. Of the mothers, 2 (4%) had a lower secondary school certificate (= 1), 11 (21%) had a middle secondary school certificate (= 2), 3 (6%) had a qualification for university of applied sciences (= 3), and 23 (43%) had a general qualification for university entrance (= 4). Thus, mother's mean level of education was 3.21 (*SD* = 1.03). Parents of child participants provided written informed consent prior to participation. Children who participated received a 15 € gift card, teachers received a 2.50 € gift card for every child they evaluated (15 € maximum), and mothers who answered the questionnaire received a 7 € gift card.

### Procedure

In summer of 2010, fifth graders participated at two group-sessions (up to 10 children) in rooms of the university. Each session lasted about 2 h and consisted of two parts (computer lab and seminar room) separated by a 10 min break. Questionnaires and standardized tests were administered in group sessions, limited to 10 children per session. The first session included the nonverbal intelligence test, the mathematics achievement test, and questionnaires. In the second session, reading and writing skills and further questionnaires were administered because the present study was part of a larger project on the relations between self-regulation and school achievement. Teachers and mothers answered paper-and-pencil questionnaires at home.

### Materials

#### Assessment of school achievement

In order to measure school achievement, grades as well as standardized reading, writing, and mathematics tests were assessed. German and mathematics grades were assessed by teachers' reports. Grades were based on children's classroom work and grades of class examinations in the first half of fifth grade (i.e., fifth grade midterm report). School grades were recoded in a way such that a higher score indicated higher school achievement (i.e., 1 = not sufficient/fail to 6 = very good). According to the German curriculum, German grades reflect, besides reading and writing skills, language proficiency (e.g., understanding the meaning of texts and reflection of language use) as well as communication and speech competencies (e.g., presentation of texts, written and oral expression; e.g., Ministerium für Kultus, Jugend und Sport Baden-Württemberg, 2004). Basic reading skills were assessed by measuring reading speed using the *Salzburger Reading-Screening for 5th to 8th graders* (Auer et al., 2008). Writing skills were measured with the *Hamburger Writing Test* (May, 2007), which consists of a text with mistakes to be corrected. This test assesses the number of corrected words and punctuation marks and provides an individual profile of orthography strategies. The mathematics subtests *numerical comprehension*, *calculation*, and *quantities* from the *Hamburger school achievement test for 4th and 5th graders* (Mietzel and Willenberg, 2000) was used in order to assess children's mathematics performance. To avoid influences of confounding variables (e.g., stereotype threat) reading, writing, and mathematics tests were conducted in a standardized manner, following the instructions of the manuals. As aggregated measures combining grades and standardized school achievement tests are more valid measures than separate measures (e.g., teachers' perceptions of children's characteristics can be related with school grades; Mullola et al., 2010), correlations were computed to test whether grades and test scores are significantly related. Pearson correlations showed significantly positive correlations of German grades to reading skills (*r* = .33, *p* < .05) and to writing skills (*r* = .37, *p* < .01) and between test performance in mathematics and mathematics grades (*r* = .48, *p* < .01). Test scores and school grades were standardized by computing z-scores and mean scores were computed for German and mathematics achievement. Accordingly, reading and writing skills and German grades were averaged into a German achievement score. Mathematics test performance and mathematics grades were averaged into a mathematics achievement score.

#### Assessment of self-regulation

In order to assess individual differences in behavior regulation, the German version of the widely used, reliable, and valid *Self-Control Scale* (Tangney et al., 2004) from Bertrams and Dickhäuser (2009) was administered. Class teachers answered the 13 items on a 5-point scale (1 = *not at all to* 5 = *very much*), e.g., “The child has a hard time breaking bad habits.”. Reliability analysis revealed a Cronbach's α of .94 in the present study.

Strategies of emotion regulation (i.e., problem- and emotion-oriented strategies) were measured using the *Questionnaire for the Measurement of Stress and Coping in Children and Adolescents* (SSKJ 3-8) (Lohaus et al., 2006). In this questionnaire, children are asked to think of a situation in which they have problems doing their homework. Children answered the items on a 5-point rating scale (from 1 = *never* to 5 = *always*) by indicating how often they use problem-oriented strategies (6 items; e.g., “I try to think of different ways to solve it.”) and emotion-oriented strategies (6 items, e.g., “I tell myself it doesn't matter.”) to cope with their emotions. Reliability analyses revealed a Cronbach's *α* of .80 for problem-oriented strategies and a Cronbach's *α* of .75 for emotion-oriented strategies.

#### Assessment of intelligence

In order to assess nonverbal intelligence, the short version of the *CFT 20-R* (Weiß, 2006) was administered. Sum scores were transformed into age-standardized IQ scores.

### Data analysis

Pearson correlations were computed to investigate associations of intelligence, age, and mother's level of education with self-regulation (i.e., behavior regulation, emotion regulation) and school achievement (i.e., German and mathematics achievement). Multivariate analyses of covariance (MANCOVAs) were computed in order to test gender differences in school achievement (i.e., German and mathematics achievement) and self-regulation (i.e., emotion and behavior regulation). Mediation models were tested by using the bootstrapping method by Preacher and Hayes (2008). Besides the fact that a bootstrapping approach is especially suitable for small sample sizes, this procedure has two strengths compared to conventional methods of mediation tests. First, multiple mediators are tested in the same model at the same time. Second, using bootstrapping avoids the assumption of a normal distribution of the indirect effects. For estimating point estimates, 5000 bootstrap samples were drawn and, for the indirect effects, 95% confidence intervals were used. A *post-hoc* power analysis was conducted to analyze, if the sample size was big enough to detect significant mediation effects (Faul et al., 2007)^1^.

## Results

Descriptive statistics are shown in Table 1. In general, boys and girls in the sample had good school achievement, as shown by their grades as well as standardized reading, writing, and mathematics tests. On average, teachers rated children's behavior regulation as high. Overall, boys and girls rated themselves as using problem-oriented strategies more often than emotion-oriented strategies. Children's nonverbal intelligence and mothers' level of education were slightly above average.

**Table 1:** **Descriptive statistics**.

**Measure**	**Boys**				**Girls**			
	**Min**	**Max**	***M***	***SD***	**Min**	**Max**	***M***	***SD***
**SCHOOL ACHIEVEMENT**								
German grade	2.00	5.10	3.97	0.80	3.00	6.00	4.48	0.72
Mathematics grade	3.00	6.00	4.63	0.73	2.00	5.80	4.44	0.74
Reading (SLS 5–8)	70.00	135.00	98.79	19.39	70.00	139.00	105.85	15.19
Writing (HSP 5–9)	0.00	69.00	47.37	16.58	23.00	70.00	53.00	9.75
Mathematics (HST 4/5)	15.00	99.00	66.21	23.68	8.00	96.00	56.65	25.29
**BEHAVIOR REGULATION**								
Behavior regulation (SCS-K-D)	1.38	4.38	3.03	0.86	1.23	4.92	3.64	0.79
**EMOTION REGULATION (SSKJ)**								
Problem-oriented strategies	2.00	5.00	3.68	0.88	1.33	5.00	3.60	0.85
Emotion-oriented strategies	1.00	3.50	2.02	0.65	1.00	4.17	2.00	0.85
**COVARIATES**								
Intelligence (CFT 20-R)	86.00	139.00	110.00	12.81	84.00	139.00	107.94	14.80
Education mother	2.00	4.00	3.80	0.63	1.00	4.00	3.00	1.07

*N* = 53, *N* (boys) = 19, *N* (girls) = 34, *N* (Education mother) = 39; German and mathematics grades were recoded: 1 = *not sufficient/fail* to 6 = *very good*. SLS 5–8 = Salzburger Reading-Screening for 5th to 8th graders; reading quotient score with *M* = 100, *SD* = 15. HSP 5–9 = Hamburger Writing-Test; *T*-values standardized for 5th graders. HST 4/5 = mathematics subtests of the Hamburger school achievement test for 4th and 5th graders; percentile ranks. SCS-K-D = German adaptation of the short version of the Self-Control Scale. SSKJ = Questionnaire for the measurement of stress and coping in children and adolescents. Intelligence = nonverbal intelligence; CFT 20-R = *Basic Intelligence Scale;* age-standardized IQ scores. Education mother = mother's level of education.

Pearson correlations revealed that age was significantly negatively correlated with intelligence and German achievement. Perhaps older children had lower nonverbal IQ and academic abilities because they already had to repeat school grades. Nonverbal intelligence correlated significantly and positively with German and mathematics achievement. No significant relations were found between mother's level of education and self-regulation (i.e., behavior regulation, problem- and emotion-oriented strategies of emotion regulation) or school-achievement variables (i.e., German and mathematics achievement) (see Table 2). Consequently, age and intelligence were entered as control variables in further analyses.

**Table 2:** **Pearson correlation matrix**.

	**1**	**2**	**3**	**4**	**5**	**6**	**7**	**8**
1. Age	−	−.45^**^	−.21	−.18	−.03	.00	−.32^*^	−.12
2. Intelligence		−	.37^*^	.14	−.04	−.04	.29^*^	.44^**^
3. Education mother			−	.07	−.02	.02	.06	.19
4. Behavior regulation				−	.05	−.25^+^	.58^**^	.35^*^
5. Problem-oriented strategies					−	−.36^**^	.02	−.08
6. Emotion-oriented strategies						−	−.36^**^	−.06
7. German achievement							−	.53^**^
8. Mathematics achievement								−

N = 53, N (Education mother) = 39;

** p < .01;

* p < .05;

+ p < .10.

Separate MANCOVAs were conducted to test gender differences in school achievement (i.e., German and mathematics achievement) and in self-regulation (i.e., behavior regulation, problem- and emotion-oriented strategies of emotion regulation). In both MANCOVAs age and intelligence were included as covariates. Using a Bonferroni adjusted alpha level of .025, the MANCOVA revealed significant gender differences in German achievement favoring girls, *F*_(1, 49)_ = 5.90, *p* = .019, η^2^ = .11, but no significant gender differences in mathematics achievement *F*_(1, 49)_ = 1.16, *p* = .287, η^2^ = .02. The MANCOVA regarding gender differences in self-regulation (i.e., behavior regulation, problem- and emotion-oriented strategies of emotion regulation) using a Bonferroni adjusted alpha level of .017, revealed a significant gender effect for behavior regulation favoring girls, *F*_(1, 49)_ = 6.65, *p* = .013, η^2^ = .12. However, there were no significant gender effects with regard to problem-oriented strategies, *F*_(1, 49)_ = .14, *p* = .706, η^2^ = .00 or emotion-oriented strategies, *F*_(1, 49)_ = .01, *p* = .918, η^2^ = .00. The means and standard deviations for school achievement and the self-regulation variables are shown in Table 3.

**Table 3:** **Summary statistics for school achievement and self-regulation variables**.

**Variable**	**Boys**		**Girls**	
	***M***	***SD***	***M***	***SD***
German achievement	−0.32	0.83	0.18	0.67
Mathematics achievement	0.21	0.83	−0.12	0.86
Behavior regulation	3.03	0.86	3.64	0.79
Problem-oriented strategies	3.68	0.88	3.60	0.85
Emotion-oriented strategies	2.02	0.65	2.00	0.85

N = 53; German and mathematics achievement are z-standardized scores; scaling behavior regulation (SCS-K-D): 5-point scale (1 = not at all to 5 = very much); scaling problem-oriented strategies and emotion-oriented strategies (SSKJ): 5-point scale (1 = never to 5 = always).

Further, we tested whether gender differences in children's school achievement were mediated by self-regulation (i.e., behavior regulation, problem and emotion-oriented strategies of emotion regulation). Therefore, two multiple mediation models were tested separately. In one model, German achievement was regarded as a dependent variable and, in the other model, mathematics achievement was regarded as a dependent variable. In both models, age and intelligence were included as control variables. Indirect effects are unstandardized coefficients, which are significant when the 95% confident interval does not contain zero.

**Figure 1:**
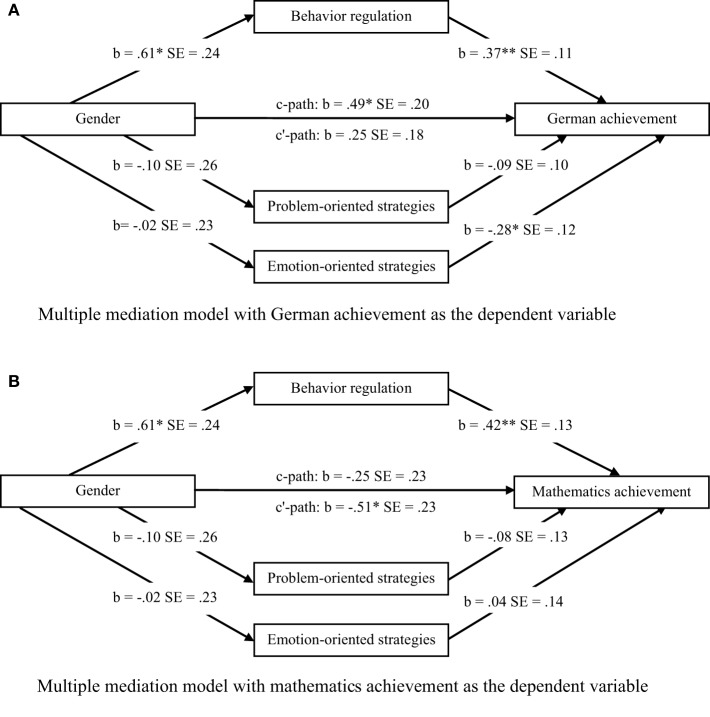
**Multiple mediation tests of the relations of gender to German and mathematics achievement mediated by behavior regulation and strategies of emotion regulation.** Multiple mediation test of the relation between gender and German achievement mediated by behavior regulation, problem-oriented strategies, and emotion-oriented strategies **(A)**. Multiple mediation test of the relation between gender and mathematics achievement mediated by behavior regulation, problem-oriented strategies, and emotion-oriented strategies **(B)**. *N* = 53; *b* = unstandardized regression coefficient, controlled for age and intelligence; ^*^p < .05; ^**^p < .01.

The relations between gender, self-regulation (i.e., behavior regulation, problem- and emotion-oriented strategies of emotion regulation), and school achievement, controlled for age and intelligence, are presented in Figure 1. Behavior regulation was significantly and positively related to German and mathematics achievement. Problem-oriented strategies were neither significantly associated with German achievement nor mathematics achievement. Emotion-oriented strategies were significantly and negatively related to German achievement but not significantly associated with mathematics achievement.

Figure 1A shows the results of the mediation model with gender as an independent variable; behavior regulation, problem-oriented strategies, and emotion-oriented strategies as mediator variables; German achievement as the dependent variable; and age and intelligence as covariates. The total effect *c* was significant, while the direct effect *c'* was non-significant. Behavior regulation significantly mediated the relation between gender and German achievement (indirect effect = .226, *SE* = .116, 95% CI [.056, .541]). Behavior regulation was a significant mediator because its 95% confidence interval did not contain zero. Neither problem-oriented strategies nor emotion-oriented strategies were significant mediators (for problem-oriented strategies: indirect effect = .009, *SE* = .036, 95% CI [−.037, .126]; for emotion-oriented strategies: indirect effect = .007, *SE* = .063, 95% CI [−.119, .144]; see Figure 1A).

Figure 1B shows the results of the mediation model with gender as an independent variable; behavior regulation, problem-oriented strategies and emotion-oriented strategies as mediator variables; mathematics achievement as the dependent variable; and age and intelligence as covariates. The total effect *c* was not significant, whereas the direct effect *c*′ was significantly negative. This means, there was no significant gender difference in mathematics achievement (total effect *c*) but, when self-regulation variables were entered in the model, there was a significant direct effect (*c*′) of gender on mathematics favoring boys. Thereby, there was a significant indirect effect of gender on mathematics achievement through behavior regulation (indirect effect = .258, *SE* = .142, 95% CI [.057, .611]). Hence, there was a suppression effect of behavior regulation on the relation between gender and mathematics achievement. Neither the indirect effect of problem-oriented strategies nor the indirect effect of emotion-oriented strategies were significant (for problem-oriented strategies: indirect effect = .008, *SE* = .010, 95% CI [−.051, .130]; for emotion-oriented strategies: indirect effect = −.001, *SE* = .026, 95% CI [−.079, .038]; see Figure 1B).

## Discussion

As hypothesized, the present study revealed that German achievement was higher for girls than for boys. There were no gender differences in mathematics achievement. These results are consistent with the results of some studies in the literature, which have also found higher achievement in German or in other language subjects (e.g., English) by girls but no significant gender differences in mathematics achievement (e.g., Spinath et al., 2010; Kuhl and Hannover, 2012). Extending previous research, we investigated gender differences in German and mathematics achievement taking children's motivation and ability for emotion and behavior regulation into account.

The results of the present study revealed that gender differences in German achievement were explained by gender differences in behavior regulation. This finding emphasizes the central function of behavior regulation for German achievement in general as well as the function of behavior regulation for gender differences in German achievement. The interpretation of the results regarding mathematics achievement is more complicated. There was no conventional mediation effect of behavior regulation on the relation between gender and mathematics achievement. Surprisingly, an interesting suppression effect occurred. There was a significant indirect effect of behavior regulation by gender on mathematics achievement. This means that the mathematics achievement of boys is underestimated when analyses do not control for behavior regulation.

The suppression effect could be a reason for the inconsistent findings regarding gender differences in mathematics achievement. The gender difference in mathematics achievement favoring boys is not found when analyses do not control for behavior regulation because girls' higher behavior regulation and the positive effect of behavior regulation on mathematics achievement cancel each other out. This finding could explain why some studies find gender differences in mathematics achievement whereas others do not, as shown in the overview by Hannover and Kessels (2011). There might be other variables that moderate the indirect effect of gender on mathematics achievement. For instance, if girls are confronted with negative stereotypes about females' mathematics achievement, their mathematics achievement worsens (e.g., Keller and Dauenheimer, 2003). A recent study by Galdi et al. (2013) has shown that even when girls are not aware of the mathematics-gender stereotype, automatic associations consistent with the stereotype may hinder girls' mathematics achievement. Hence, for girls with strong negative stereotypes about their mathematics achievement or with the presence of stereotype-consistent automatic associations, behavior regulation might be less strongly related to girls' mathematics achievement in comparison to girls with less negative gender stereotypes. In this case, gender differences in mathematics achievement, favoring boys can be found. Without the presence of stereotypes or stereotype-consistent automatic associations, no gender differences in mathematics achievement would be found because of the suppression effect of behavior regulation. In contrast to former studies, in addition to behavior regulation, we examined the role of emotion regulation on gender differences in school achievement. The present study revealed that strategies of emotion regulation (i.e., problem- and emotion-oriented strategies of emotion regulation) did not mediate the relation between gender and school achievement. As *post-hoc* power analyses revealed low power for detecting small and medium effects, future studies with larger samples and higher power may find significant mediation effects of emotion regulation strategies. Nevertheless, the present study revealed a significant and negative relation between the use of emotion-oriented strategies of emotion regulation and German achievement. This result suggests that children who tend to engage in active coping are more likely to show higher German achievement than children who tend to disengage mentally and behaviorally from stressful school-related situations (e.g., a lot of homework).

### Strengths and limitations

Although the sample size was rather small and children came from a rather homogeneous middle-class socio-economic background, analyses revealed significant gender differences in behavior regulation and German achievement. For instance, gender accounted for a substantial amount of variance in behavior regulation (12%) and German achievement (11%). However, future research using larger and more diverse samples is desirable in order to be able to generalize the findings of the present study to larger populations. Furthermore, emotion regulation was assessed by children's self-reports only. Further studies should include a direct measure of emotion regulation as well as a multiple-measure strategy that takes also other strategies of emotion regulation into account (e.g., reappraisal; Gross and Thompson, 2007). In addition, the present study relied on class teachers' reports for the assessment of children's behavior regulation. Ideally, to measure behavior regulation, direct and multiple-measure strategies should be used. It should also be noted that school grades are teacher evaluations, too. In order to take these limitations into account school achievement was assessed by school grades (i.e., mid-term report grades in German and mathematics) and by standardized achievement tests. Moreover, children's self-regulation (i.e., behavior regulation, emotion regulation) was assessed by teacher report and a self-report measure.

### Theoretical implications

In line with previous results, the present study revealed that German achievement and the motivation and ability for behavior regulation was higher for girls than for boys. Moreover, indirect effects of gender on German and mathematics achievement were mediated by children's behavior regulation, but not by strategies of emotion regulation. Furthermore, mediation analyses indicated that mathematics achievement was higher for boys than for girls. However, gender differences in mathematics achievement were canceled out because of girls' higher motivation and ability for behavior regulation that was positively associated with mathematics achievement. Hence, further studies analyzing gender differences in mathematics achievement should consider the possibility that the mathematics achievement of boys may be underestimated when not controlling for behavior regulation. Further studies should investigate whether variables such as stereotype threat moderate relations between gender, behavior regulation, and mathematics achievement. Moreover, as culture influences the development of self-regulation (Trommsdorff, 2009; Heikamp et al., 2013), longitudinal studies are needed to draw causal conclusions concerning the effect of socialization in different contexts (e.g., culture, family, school) on the development of gender differences in self-regulation and school achievement.

### Conflict of interest statement

The authors declare that the research was conducted in the absence of any commercial or financial relationships that could be construed as a potential conflict of interest.

## Footnotes

^1^For the statistical power analyses the sample size of 53, the number of predictors of 6, the alpha level of *p* < .05, and Cohen's (1988) criteria of effect sizes (small [*f*^2^ = .02], medium [*f*^2^ = .15], and large [*f*^2^ = .35]) were used. The *post-hoc* analyses revealed that the statistical power for the mediation analyses was .09 and .47 to detect small and medium effects, whereas it was .87 for for detecting large effects. Hence, there was a high power at the high effect size level, but a low power at the medium and small effect size level.
